# Migraine With Aura Is Related to Delayed Motor Control Reaction and Imbalance Following External Perturbations

**DOI:** 10.3389/fneur.2021.755990

**Published:** 2021-11-08

**Authors:** Gabriela F. Carvalho, Kerstin Luedtke, Carina F. Pinheiro, Renato Moraes, Tenysson W. Lemos, Marcelo E. Bigal, Fabiola Dach, Debora Bevilaqua-Grossi

**Affiliations:** ^1^Department of Health Sciences, Ribeirão Preto Medical School, University of São Paulo, Ribeirão Preto, Brazil; ^2^Department of Physiotherapy, Institute of Health Sciences, University of Luebeck, Luebeck, Germany; ^3^Laboratory of Pain Research, Institute of Physiotherapy and Health Sciences, The Jerzy Kukuczka Academy of Physical Education, Katowice, Poland; ^4^Biomechanics and Motor Control Lab, School of Physical Education and Sport of Ribeirão Preto, University of São Paulo, Ribeirão Preto, Brazil; ^5^Ventus Therapeutics, Montreal, QC, Canada; ^6^Department of Neurosciences and Behavioral Sciences, Ribeirão Preto Medical School, University of São Paulo, Ribeirão Preto, Brazil

**Keywords:** migraine disorders, aura, postural control, clinical evaluation, posturography

## Abstract

**Background:** It is evidenced that migraineurs present balance deficits. However, the balance recovery following unexpected ground perturbations, which reflect conditions of everyday activities, has not been investigated in this population.

**Aim:** We aimed to assess the reactive postural responses among patients with migraine with and without aura, chronic migraine, and controls. We further aimed to assess the factors associated with greater self-report of falls.

**Methods:** Ninety patients diagnosed by headache specialists were equally classified into three migraine subgroups according to the presence of aura and chronic migraine. Thirty controls were also recruited. All participants underwent the motor control test (MCT) and adaptation test (ADT) protocols of dynamic posturography tests (EquiTest®, NeuroCom, USA). Clinical and headache features and information on falls in the previous year, fear of falling, and vestibular symptoms were also assessed.

**Results:** Patients with aura presented a greater sway area in most of the MCT conditions than the other three groups (*p* = 0.001). The aura group also presented delayed latency responses after perturbations compared with controls and patients without aura (*p* < 0.03). In the ADT, a greater sway area was observed in patients with aura than in groups without aura, chronic migraine, and controls (*p* < 0.0001). The MCT and ADT sway area, the frequency of aura, and the fear of falling explained 46% of the falls in the previous 12 months.

**Conclusion:** Patients with aura exhibited greater delay and sway area after unexpected ground perturbations than controls and other migraine subgroups, which are related to the reported number of falls.

## Introduction

Postural control depends on the integrity of multiple complex mechanisms to achieve (1) upright stability under different sensory conditions, (2) coordination and balance during voluntary movements, and (3) motor reactions under external destabilizing conditions, such as a slip or push (1, 2). For upright stability, recent studies have demonstrated alterations among patients with migraine in quiet standing conditions, including firm and foam surfaces, with open and closed eyes (3–6). Performance and balance deficits during voluntary movements are also verified in this population in contrast to controls during daily activities (5, 7, 8). While stability during upright standing is impaired in migraine with aura and chronic migraine compared with patients without aura (5, 6, 9), their performance during voluntary movements does not differ among the migraine subtypes (7).

Despite these findings associating migraine with lower functionality and balance changes, aspects related to balance recovery after sudden external perturbations remain largely unexplored in this population. The ability to perform a successful reactive response following an unexpected perturbation is crucial to prevent a fall (10). The performance of adequate corrective motor responses requires an adequate integration among neural, sensory, and musculoskeletal systems (11, 12).

The detection of postural deficits following a perturbation is essential to tailor rehabilitation programs (13), once they reflect conditions underlying everyday activities that involve feedback-based postural reactions, such as slips or trips (14). Accordingly, we aimed to assess the reactive postural responses among patients with migraine with and without aura, chronic migraine, and healthy controls. Furthermore, we aimed to determine which factors are associated with a greater number of self-reported falls in the last year. Based on previous evidence of postural control impairment among migraineurs (3–8), we hypothesized that deficits in reactive postural responses would also be verified in all migraine subtypes in contrast to controls.

## Methods

### Participants

This cross-sectional study was approved by the Investigation Review Board of the University Clinical Hospital of Ribeirão Preto (process number: 15572/2016). All included participants provided written informed consent before enrollment in the study. The sample of migraineurs was recruited in a tertiary headache center and the local community, with migraine diagnosis made by neurologists, following the criteria established by the International Headache Society in the third edition of the International Classification of Headache Disorders (15). Patients with migraine (*n* = 90) were stratified equally into three subgroups according to the presence of aura (migraine with and without aura, MA and MoA, respectively) and frequency of attacks over 15 days within a month (chronic migraine, CM). A group of healthy participants (CG, *n* = 30) composed of family members of the patient or hospital staff were also recruited.

We included women from 18 to 55 years old. Migraine participants had to have a minimum of 3 days of headache per month within the last 3 months. Controls were included if they reported no primary headache, and any secondary headache with occurrence greater than two times within the last 6 months. Exclusion criteria encompassed the diagnosis of any rheumatic, neurologic, cardiovascular, or vestibular pathology [such as neuritis, benign paroxysmal positional vertigo (BPPV) or Ménière's disease], as well as pregnancy or any chronic pain condition. Abnormal neurological examination results and patients with any concomitant primary or secondary headaches (i.e., tension-type headache or medication-overuse headache) were also excluded. For the homogeneity of the sample, patients with aura had to be diagnosed with typical aura, and therefore, we did not include the diagnosis of brainstem aura, hemiplegic migraine, or retinal migraine. Furthermore, if patients reported a migraine attack on the appointment day, the evaluation was rescheduled to a headache-free day.

### Procedures

Participants who met the inclusion and exclusion criteria answered a questionnaire recording age, height and weight, migraine frequency, onset, intensity and duration, medication intake, presence of vestibular symptoms, and the number of falls within the last 12 months. Falls were defined according to the World Health Organization (WHO) (16). Furthermore, participants were instructed to answer the Falls Efficacy Scale-International (FES-I), which measures the level of concern with fall occurrence during functional daily living activities (17). The FES-I scores range between 16 and 64, and higher scores indicate greater concern levels and fear of falling.

Afterward, the participants were referred to an examiner blinded toward the study groups, who guided the physical exam in the computerized dynamic posturography equipment (EquiTest®, NeuroCom, OR, USA). The participants were instructed to step over the platform, positioning the feet apart at a standardized distance, according to their height as described by the guide brochure of the manufacturer (18). They were secured by an overhead harness, which would prevent falls without limiting body movement. Participants performed the motor control (MCT) and adaptation (ADT) tests in the EquiTest®.

The MCT consisted of assessing the motor reactions of the participants following an unexpected translation of the support surface in forward and backward directions in three levels (small, medium, and large). For both forward and backward directions, the small translation consisted of 0.7° of equivalent sway for 250 ms, the medium translation has 1.8° of equivalent sway for 300 ms, and the large translation has 3.2° of equivalent sway for 400 ms (18). Each excursion was repeated three times. The mean latency was determined by the elapsed time in milliseconds (ms) between the onset of the support translation until the active sway response of the participant. Furthermore, a composite latency was assessed considering all the test conditions. This test is considered a valid and reliable measure (19–21), with no significant learning effect (20). It is also highly correlated with motor performance and falls occurrence, and has been used to assess several neurologic and vestibular conditions (1, 14, 22).

The ADT also assessed motor reactions, but following abrupt platform rotations to the direction of toes up and down, with an amplitude of 8° and duration of 400 ms. Participants performed five trials for each direction, and the mean of the trials and conditions was considered. The mean sway energy (ranging from 0 to 200) for toes up and down was measured. This outcome is calculated by the Equitest® software, based on the weighted sum of the RMS velocity and acceleration of the anteroposterior center of pressure (CoP) displacement. Lower scores reflect better adaptation on minimizing sway after the support surface rotation (18).

Both MCT and ADT tests started after random delays lasting between 3 and 5 s to prevent movement prediction and consider the response based on the automatic postural control system (18). Furthermore, we also considered, for both tests, the CoP sway area as an outcome, which comprised 90% of the displacement ellipse (cm^2^) in the MCT and ADT tests. This outcome reflected the body instability induced by the support surface excursion during the attempt to recover balance. Based on the exported raw CoP data obtained by the force plates, the sway area was calculated using the MATLAB 2019a software (23).

### Statistical Analysis

The sample size for this study was calculated based on a pilot study with 10 patients with MoA and 10 controls, considering a difference between groups of 12 ms in the composite latency score of the MCT test. A minimum of 28 subjects was required to detect differences between groups with an effect size of 0.8, alfa of 5%, and 90% of power. Mean, standard deviations, or 95% confidence interval (CI) were calculated to present clinical characteristics of each group. Variables with normal distribution (non-significant Kolmogorov–Smirnov test) were compared through analysis of variance (ANOVA) for independent samples with Bonferroni *post-hoc* test. The distribution of nominal data was presented through percentages, and groups were compared through Chi-squared tests.

All the MCT and ADT test outcomes were analyzed through multiple generalized linear models considering all the test conditions and further corrected for multiplicity by Bonferroni correction. Furthermore, a multiple linear regression using backward elimination was calculated to explain the variability of the number of reported fall events in the last 12 months. The following clinical and motor control variables were included in the model: migraine onset, frequency of attacks, frequency of aura, fear of falling scores (FES-I), intake of prophylactic medication, ADT sway area, MCT composite latency, and sway area during the medium and large backward and forward translations of the MCT. A minimum of 10 participants per variable was considered in the linear regression analysis (24).

## Results

Table 1 presents the demographic data of the participants. Differences among groups were found regarding prophylactic medication intake (*x*^2^ = 15.23*, p* = 0.002), migraine frequency (*F* = 141.17, *p* < 0.0001), self-report of ictal (*x*^2^ = 54.98, *p* < 0.001) and interictal vestibular symptoms (*x*^2^ = 13.81, *p* < 0.003), self-report of falls within the last 12 months (*F* = 5.92, *p* = 0.001), and FES-I scores (*F* = 10.82, *p* < 0.0001). No differences between groups were found regarding age, BMI, migraine onset, duration, or intensity.

**Table 1:** Mean (SD) and percentages (%) of the sample demographic characteristics.

	**Control group**	**Migraine without aura**	**Migraine with aura**	**Chronic migraine**	***Statistical result***
Age (years)	31.3 (9.3)	32.5 (8.7)	32.2 (8.3)	34.6 (10.0)	*F* = 0.68, *p* = 0.556
BMI (kg/cm^3^)	24.9 (4.1)	24.1 (3.6)	24.5 (4.2)	23.8 (2.9)	*F* = 0.51, *p* = 0.67
Migraine onset (years)	–	15.5 (7.8)	18.0 (9.2)	18.0 (10.9)	*F* = 0.73, *p* = 0.48
Migraine frequency (attacks/month)	–	7.3 (3.3)^‡^	7.6 (2.9)^‡^	23.3 (5.8)	***F*** **=** **141.17**, ***p*** **<** **0.0001**
Aura frequency (attacks/month)	–	0 (0)	4.10 (2.54)^†^	1.87 (4.07)^†^	***F*** **=** **16.46**, ***p*** **<** **0.0001**
Migraine duration (h)	–	17.8 (20.5)	34.0 (29.4)	26.2 (27.5)	*F* = 2.87, *p* = 0.06
Migraine intensity (NRS: 0–10)	–	7.4 (1.3)	7.6 (1.9)	8.1 (1.7)	*F* = 1.15, *p* = 0.31
Prophylactic medication intake (%)	10%	30%	40%	56.7%	***x**^**2**^* **=** **15.23*****, p*** **=** **0.002**
Interictal vestibular symptoms (%)	13%	37%	57%	50%	***x***^**2**^ **=** **13.81**, ***p*** **<** **0.003**
Ictal vestibular symptoms (%)	0%	60%	87%	77%	***x***^**2**^ **=** **54.98**, ***p*** **<** **0.001**
Number of falls (last 12 months)	0.3 (0.5)	1.4 (2.4)	4.6 (5.8)^*^	4.4 (7.2)^*^	***F*** **=** **5.92**, ***p*** **=** **0.001**
Falls efficacy scale (FES-I)	20.1 (4.5)	23.7 (5.5)	27.5 (4.9)^*^	27.3 (7.8)^*^	***F*** **=** **10.82**, ***p*** **<** **0.0001**

*SD, standard deviation; BMI, body mass index; NRS, numeric rating scale (0–10). Bonferroni post-hoc*:

* *p <0.02 vs. control group*.

† *p <0.03 vs. migraine without aura group*.

‡ *p <0.0001 vs. chronic migraine group. Bold expresses significant results*.

**Figure 1:**
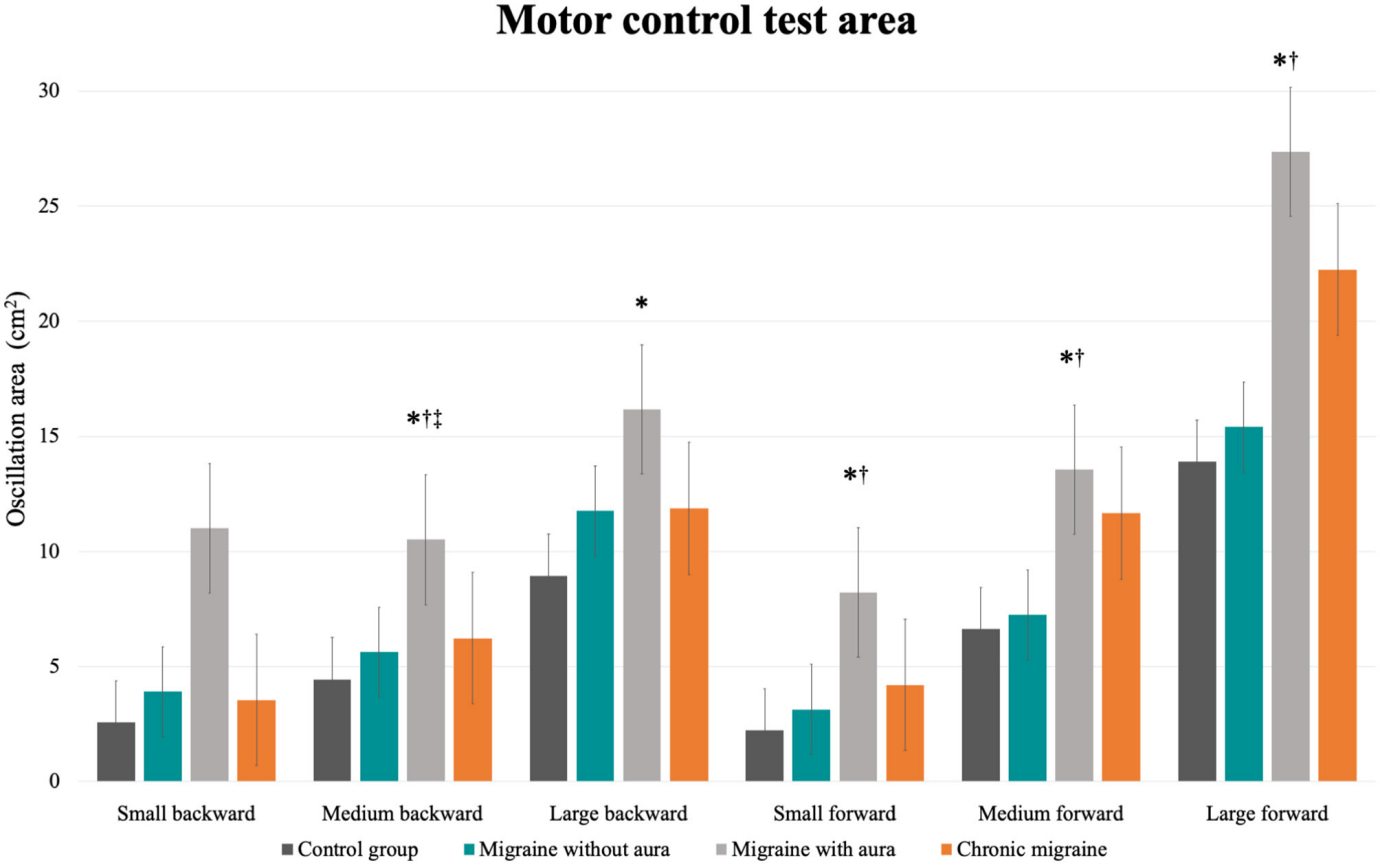
Mean and standard errors of the oscillation area (cm^2^) of each condition of the motor control test among patients with migraine with and without aura, chronic migraine, and controls. *p < 0.01 vs. control group; †*p* < 0.04 vs. migraine without aura; ^‡^*p* = 0.03 vs. chronic migraine.

**Figure 2:**
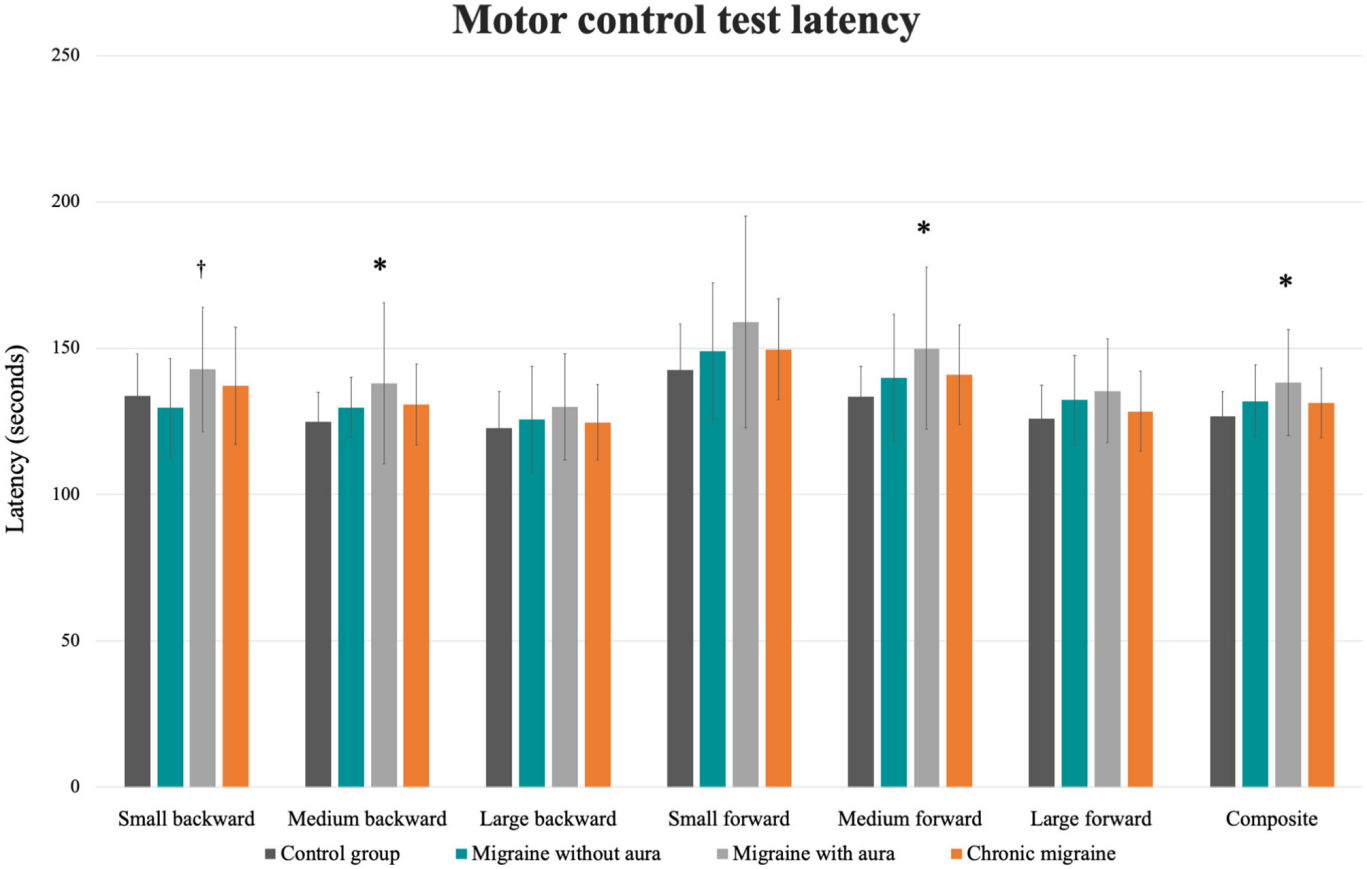
Mean and standard errors of the latency (s) of each condition of the motor control test among patients with migraine with and without aura, chronic migraine, and controls. *p < 0.01 vs. control group; †*p* < 0.04 vs. migraine without aura.

In the MCT test, patients with migraine with aura presented greater sway area after medium backward perturbation than the other three groups [MA: 10.51 (8.43 to 12.59) vs. CG: 4.44 (2.36 to 6.52), vs. MoA: 5.63 (3.55 to 7.71), vs. CM: 6.22 (4.14 to 8.30); *F* = 6.34, *p* = 0.001] and after large backward perturbation than controls [MA: 16.17 (13.45 to 18.90) vs. CG: 8.93 (6.20 to 11.65); *F* = 4.71, *p* = 0.004]. Following the forward perturbations, patients with aura presented a greater sway area than controls and migraineurs without aura in the small [MA: 8.23 (5.96 to 10.49) vs. CG: 2.21 (−0.04 to 4.48), vs. MoA: 3.12 (0.86 to 5.39); *F* = 5.38, *p* = 0.002], medium [MA: 13.54 (10.43 to 16.66) vs. CG: 6.63 (3.51 to 9.74), vs. MoA: 7.23 (4.12 to 10.35); *F* = 4.59, *p* = 0.004], and large amplitudes [MA: 27.37 (21.55 to 33.48) vs. CG: 13.90 (7.79 to 20.02), vs. 15.40 (9.28 to 21.51); *F* = 4.10, *p* = 0.008]. The aura group also presented delayed latency responses after perturbation in contrast to controls for medium backward [MA: 138.00 (131.84 to 144.15) vs. CG: 124.78 (118.63 to 130.93); *F* = 3.07, *p* = 0.03] and medium forward [MA: 150.00 (142.70 to 157.30) vs. CG: 133.53 (126.23 to 140.83); *F* = 3.38, *p* = 0.02] perturbations, and composite latency [MA: 138.37 (133.60 to 143.14) vs. CG: 126.67 (121.90 to 131.43); *F* = 3.99, *p* = 0.01]. In contrast to migraineurs without aura, delayed latency response was verified in patients with aura for the small backward perturbation [MA: 142.77 (136.70 to 149.40) vs. MoA: 133.83(127.20 to 140.47); *F* = 2.74, *p* = 0.04]. These results are presented in Table 2 and Figures 1, 2.

**Table 2:** Mean and 95% CI of sway area and latency during the motor control test (MCT) and adaptation test (ADT) among migraine groups and controls.

		**Control group**	**Migraine without aura**	**Migraine with aura**	**Chronic migraine**
MCT area (cm^2^)	Small backward	2.57 (−3.02 to 8.17)	3.90 (−2.69 to 9.50)	11.02 (5.42 to 16.62)	3.54 (−2.05 to 9.14)
	Medium backward	4.44 (2.36 to 6.52)	5.63 (3.55 to 7.71)	10.51 (8.43 to 12.59)^*^^†^^,^^‡^	6.22 (4.14 to 8.30)
	Large backward	8.93 (6.20 to 11.65)	11.75 (9.03 to 14.48)	16.17 (13.45 to 18.90)^*^	11.86 (9.14 to 14.59)
	Small forward	2.21 (−0.04 to 4.48)	3.12 (0.86 to 5.39)	8.23 (5.96 to 10.49)^*^^†^	4.20 (1.94 to 6.47)
	Medium forward	6.63 (3.51 to 9.74)	7.23 (4.12 to 10.35)	13.54 (10.43 to 16.66)^*^^†^	11.66 (8.54 to 14.77)
	Large forward	13.90 (7.79 to 20.02)	15.40 (9.28 to 21.51)	27.37 (21.55 to 33.48)^*^^†^	22.24 (16.31 to 28.36)
MCT latency (s)	Small backward	133.83 (127.20 to 140.47)	129.67 (123.03 to 136.30)	142.77 (136.70 to 149.40)^†^	137.33 (130.70 to 143.97)
	Medium backward	124.78 (118.63 to 130.93)	129.83 (123.68 to 135.98)	138.00 (131.84 to 144.15)^*^	130.83 (124.68 to 136.98)
	Large backward	122.67 (117.02 to 128.31)	125.63 (119.98 to 131.28)	130.00 (124.35 to 135.64)	124.67 (119.02 to 130.31)
	Small forward	142.50 (133.66 to 151.34)	149.00 (140.16 to 157.84)	159.00 (150.16 to 167.84)	149.67 (140.82 to 158.51)
	Medium forward	133.53 (126.23 to 140.83)	140.00 (132.69 to 147.30)	150.00 (142.70 to 157.30)^*^	141.00 (133.69 to 148.30)
	Large forward	126.00 (120.67 to 131.32)	132.33 (127.01 to 137.66)	135.50 (130.17 to 140.82)	128.50 (123.17 to 133.82)
	Composite	126.67 (121.90 to 131.43)	132.00 (127.22 to 136.77)	138.37 (133.60 to 143.14)^*^	131.30 (126.53 to 136.07)
ADT area (cm^2^)		10.18 (7.66 to 12.7)	13.15 (10.63 to 15.67)	19.04 (16.52 to 21.56)^*^^†^^,^^‡^	13.79 (11.26 to 16.31)
ADT sway energy		58.73 (54.97 to 62.49)	57.86 (54.1 to 61.62)	59.33 (55.57 to 63.09)	58.85 (55.09 to 62.61)

*MCT, motor control test; ADT, adaptation test*.

* *p <0.01 vs. control group*;

† *p <0.04 vs. migraine without aura*;

‡ *p = 0.03 vs. chronic migraine*.

**Figure 3:**
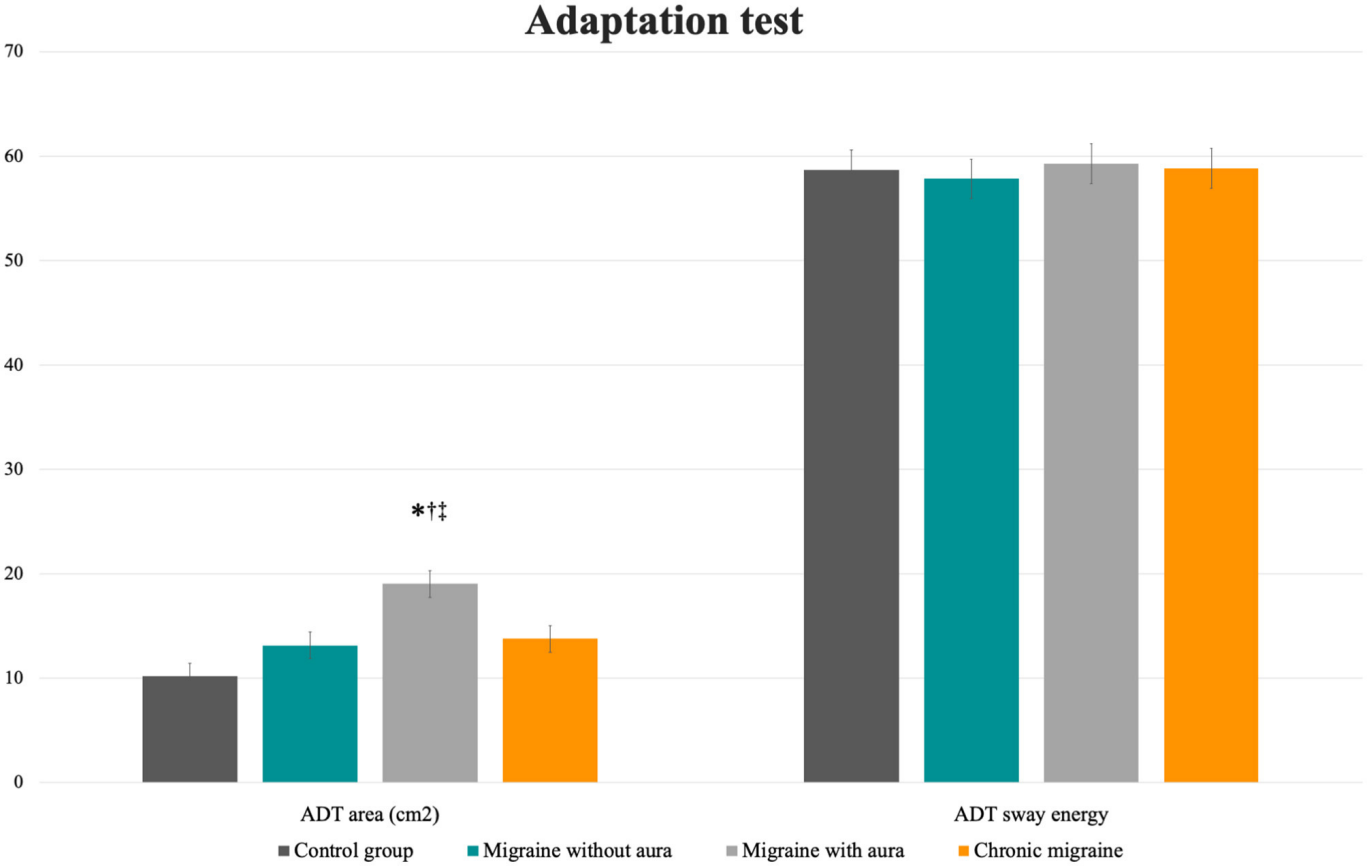
Mean and standard errors of the area (cm^2^) and sway energy of the adaptation test among patients with migraine with and without aura, chronic migraine, and controls. *p < 0.01 vs. control group; †*p* < 0.04 vs. migraine without aura; ^‡^*p* = 0.03 vs. chronic migraine.

No differences among groups were verified for the mean sway energy for the ADT test (*F* = 0.10, *p* = 0.96). However, patients with aura presented a greater sway area following the perturbations than the groups without aura, chronic migraine, and controls [Table 2; Figure 3: MA: 19.04 (16.52 to 21.56) vs. CG: 10.18 (7.66 to 12.7), vs. MoA: 13.15 (10.63 to 15.67), vs. CM: 13.79 (11.26 to 16.31); *F* = 8.39, *p* < 0.0001].

Table 3 shows the results of the multiple linear regression. The initial model presented a significant regression equation [*F*_(11,108)_ = 9.14, *p* < 0.0001], with an *R*^2^ of 0.48. After the backward criteria for variable exclusion, the last model included five significant predictors [*F*_(5,114)_ = 19.18, *p* < 0.0001] with an *R*^2^ of 0.46. Predicted number of falls of the participants are influenced by the frequency of aura (+0.62), FES-I scores (+0.28), by the MCT medium front oscillation area (−0.19), by MCT large front oscillation area (+0.08), and by the ADT area (+0.10).

**Table 3:** Multiple linear regression for prediction of the number of falls based on clinical and motor reaction variables.

**Models**		**Unstandardized**		**Standardized**							
		**coefficients**		**coefficients**							
		***B***	**Std. error**	**Beta**	***t***	**Sig**.	***R* square**	**Adjusted *R* square**	***df***	***F***	**Sig**.
1	Constant	−8.13	3.67		−2.22	0.03	0.48	0.43	11	9.14	<0.0001
	Migraine onset	−0.06	0.04	−0.12	−1.35	0.18					
	Migraine frequency	0.09	0.05	0.16	1.92	0.06					
	Frequency of aura	0.64	0.14	0.37	4.59	<0.0001					
	Intake of prophylactic medication	−0.47	0.51	−0.07	−0.91	0.36					
	Fear of falling scores (FES-I)	0.27	0.07	0.34	3.94	<0.0001					
	MCT medium back area	−0.07	0.08	−0.09	−0.91	0.37					
	MCT large back area	0.01	0.06	0.02	0.23	0.82					
	MCT medium front area	−0.17	0.06	−0.29	−2.75	0.01					
	MCT large front area	0.07	0.03	0.26	2.46	0.02					
	MCT composite latency	0.02	0.03	0.04	0.52	0.60					
	ADT area	0.12	0.06	0.18	2.12	0.04					
9	Constant	−6.27	1.47		−4.26	<0.0001	0.46	0.43	5	19.19	<0.0001
	Frequency of aura	0.62	0.14	0.36	4.60	<0.0001					
	Fear of falling scores (FES-I)	0.28	0.06	0.36	4.53	<0.0001					
	MCT medium front area	−0.19	0.06	−0.34	−3.38	0.001					
	MCT large front area	0.08	0.03	0.29	2.85	0.01					
	ADT area	0.10	0.05	0.14	1.80	0.07					

*MCT, motor control test; ADT, adaptation test*.

## Discussion

Our results partially confirmed our initial hypothesis that altered responses following the support surface perturbation would be verified in all migraine subtypes compared with healthy controls. Increased response delay and postural sway area were observed only among patients with aura vs. controls and in some conditions compared with the remaining migraine groups. We further found that the MCT and ADT sway area, the frequency of aura, and fear of falling explained 46% of the falls in the previous 12 months.

The presence of aura seems to have a negative impact on the static balance of the migraineurs (5, 6, 9), and this is a strength of our work since most of the previous studies did not distinguish groups based on migraine subdiagnosis (4, 8, 25–29). However, for postural control during dynamic tasks such as gait, sit to stand, climb up and down the stairs, or limits of stability, comparable performance was verified among patients with and without aura and chronic migraine, with all differing from controls (6, 7). Interestingly, contrary to previous studies on static balance (5, 9), patients with chronic migraine did not show a decreased performance while reacting from external perturbations.

This specific task of external perturbation reaction involves a complex sequence of balance correcting synergies based on preprogrammed muscle patterns within the postural control networks in the central nervous system (14). The muscle synergy activation elicits a motor response combining trunk, upper, and lower limb movements to stabilize balance (30), after a triggering input from the somatosensory, visual, and/or vestibular systems (11). Peripheral and central areas, including the spinal cord, brainstem, cerebellum, basal ganglia, parietal, and frontal areas, are involved in controlling the standing balance when external perturbations are present (14, 31).

In contrast to migraineurs without aura, patients with aura often present an additional burden, including greater self-reported vestibular disorders (32, 33), depression (34), greater stroke risk, and presence of subclinical ischemic brain lesions (35). In our sample, patients with aura and chronic migraine presented a greater prevalence of vestibular symptoms, both ictally and interictally. The lack of motor control differences between chronic migraineurs and headache-free subjects suggests that the presence of vestibular symptoms in migraine may not influence balance, as previously suggested (5, 9, 26, 27).

Despite numerous neurophysiological and neuroimaging studies have evidenced somatosensory and motor dysfunctions in the migraine brain (36), the present study showed clinical alterations in the motor control reactions just in patients with aura. These findings could be related to specific neurophysiological alterations among patients with aura, such as greater motor-evoked potential amplitudes, lack of blink reflex habituation, visual and motor cortex excitability abnormalities, reduced cerebellar inhibition, and neuromuscular transmission dysfunctions (36, 37). Studies investigating the evoked potentials in patients with aura frequently reported greater neural response to any kind of sensory stimuli, which could be explained by abnormal short- and long-term adaptive processes to external stimuli (36) and can be directly influenced by the migraine phase (38). This is complemented by experimental studies, which suggest that these alterations reflect the activation of trigeminovascular nociceptors *via* cortical spreading depression (36). Despite the ongoing debate about migraine with and without aura being considered distinct disorders, our results demonstrate a specific clinical presentation of this disease subtype, possibly reflecting the observed neurophysiological alterations—which so far were considered to be subclinical.

We also found that reduced motor reaction performance, fear of falling, and aura frequency can predict the fall events reported in the previous year. These findings have a substantial relevance in the clinical setting and are in line with previous studies that highlighted the relevance of balance perturbation-based tests to assess fall risk in stroke and older adult populations (13, 30, 39). It is further known that perturbation-based balance training reduces fall risk among older adults and patients with Parkinson's disease (40). Further research is warranted to state whether specific balance rehabilitation programs should also be implemented for patients with migraine with aura, aiming to decrease the balance deterioration and fall risk, also seen in perturbation-based assessment protocols.

Our study has some limitations. The inclusion of just females in our study, does not allow for a generalization to other populations. Furthermore, our study cannot make any statements regarding etiology due to its cross-sectional design. Although all patients were pain-free during the assessment, we cannot exclude that they were pre-ictal or post-ictal. This can be considered a factor of bias, along with the group differences in the prophylactic medication intake. On the other hand, no differences were found between the group with higher attack frequency and greater medication intake (chronic migraine) in contrast to controls. Finally, it is important to point out as a limitation that our evaluation of the number of fall events within the last 12 months might not be free of recall bias, and the results should therefore be interpreted with caution. However, this is the first study to assess the responses to perturbations in different subgroups of patients with migraine, shedding light on a precise knowledge of musculoskeletal and neuromuscular deficits among this population. The characterization of meaningful and functional mechanisms of balance control, which mimic daily life sensory conditions, can potentially improve the clinical assessment and provide valuable tools for answering clinical research questions (13).

## Conclusion

Patients with migraine with aura exhibited greater response delay and sway area than controls and other migraine subgroups during the assessment of balance following unexpected ground perturbations. The imbalance after the ground perturbation along with the frequency of aura and fear of falling explained almost half of the fall events reported during the last year. These findings indicate additional comorbidities related to motor control in patients with migraine aura, and etiologies remain to be elucidated in future studies.

## Data Availability Statement

The original contributions presented in the study are included in the article, further inquiries can be directed to the corresponding author/s.

## Ethics Statement

The studies involving human participants were reviewed and approved by Investigation Review Board of the Clinics Hospital (process number: 15572/2016). The patients/participants provided their written informed consent to participate in this study.

## Author Contributions

GC, MB, and DB-G conceptualized the study. GC and CP performed the data curation. GC, TL, and RM performed the formal analysis. GC and DB-G acquired the funding. GC, KL, RM, MB, DB-G, and FD formulated the methodology. MB and DB-G supervised the study. GC wrote the original draft. GC, KL, CP, RM, MB, FD, and DB-G reviewed and edited the manuscript. All authors contributed to the article and approved the submitted version.

## Funding

Funding was provided by the Fapesp Foundation, grants 2015/18031-5 and 2017/07482-1.

## Conflict of Interest

MB was employed by company Ventus Therapeutics. The remaining authors declare that the research was conducted in the absence of any commercial or financial relationships that could be construed as a potential conflict of interest.

## Publisher's Note

All claims expressed in this article are solely those of the authors and do not necessarily represent those of their affiliated organizations, or those of the publisher, the editors and the reviewers. Any product that may be evaluated in this article, or claim that may be made by its manufacturer, is not guaranteed or endorsed by the publisher.
